# Indigenous artifacts from remote areas, used to design a lesson plan for preservice math teachers regarding sustainable education

**DOI:** 10.1016/j.heliyon.2021.e06417

**Published:** 2021-03-13

**Authors:** Niken Wahyu Utami, Suminto A. Sayuti, Jailani Jailani

**Affiliations:** a Universitas PGRI Yogyakarta, Yogyakarta, Indonesia; b Yogyakarta State University, Yogyakarta, Indonesia

**Keywords:** Sustainable education, Indigenous knowledge, Ethnomathematics, Preservice math teacher, Math lesson plan

## Abstract

In the context of sustainable education, remote areas require special treatment. However, teachers are not evenly distributed in terms of quantity and quality. Adaptable, creative, and innovative teachers are needed in remote areas. Therefore, universities must prepare preservice teachers to teach in these places. This study explores indigenous artifacts from local communities related to mathematical content that preservice teachers can adopt to design lesson plans using available resources. Data were collected through the artifacts of indigenous people in a mountainous region on the border of Yogyakarta and Central Java, Indonesia, and the math curriculum content was examined. The relational ideas of the artifacts and the math curriculum content were analyzed. Based on the results, this study shows that artifacts can be incorporated into math learning materials. Elaborating on the artifacts can potentially relate culture and math in the classroom. The artifacts contain mathematical value and are close to students' thoughts. Hence, preservice math teachers can use them to design lesson plans, particularly for math learning. By understanding artifacts in remote areas, preservice teachers will have a particular capability for preparing lesson plans relevant to students’ environment for sustainable education.

## 1 Introduction

Problems affecting sustainable development include a lack of technology and limited opportunities for an alternative perspective on problems. However, education tends to resolve these issues because it changes an individual's or the community's mindset and behavior mode. Education is the basis for unlocking human capabilities (WDR, 2018). It is also a key instrument in transforming or achieving the Sustainable Development Goals (SDGs) (UNESCO, 2017). Its objectives are to raise upright and dutiful citizens with the desire, attitude, and skills required to build a sustainable society (Chikamori et al., 2016).

Education is a solution toward achieving sustainable development. However, improper learning and educational disparities are also part of sustainable development issues in almost all countries, regions, remote areas, minorities, and also in Indonesia. Indonesia consists of various islands, hills, beaches, and numerous ethnic groups. These factors have led to a variety of characteristics, conditions, cultures, and differences in development. There are areas in the country that are categorized as remote with unique characteristics. In this research, such areas are referred to as those situated in hilly locales and near provincial or other countries’ borders.

Students in remote zones need proper education. Cajete (Lee, 2015) stated that indigenous education produces humans that contribute immensely to their communities. Learning that employs familiar situations tends to make it easier for students to solve immediate problems.

The combined efforts of indigenous and scientific knowledge permits the possibility of sustainability at various scales and in the educational sector (Hill et al., 2020; Tengo et al., 2017). One activity related to students' situation is learning materials from indigenous communities. To compile these learning materials, the teacher needs to be adaptive, innovative, and creative to suit the students' environment, including the conditions and circumstances of the facilities. The training of preservice teachers from the beginning to higher education is essential to produce quality teachers that understand people's conditions and competence in such areas. Another preparation is the ability to be adaptive, creative, and innovative in designing learning based on students' environmental conditions.

Education for preservice teachers promotes sustainability in the academic sector, representing a new paradigm for their preparation. It helps develop a curricular vision and conversation regarding teacher education's role in solving global environmental and social justice challenges (Nolet, 2009)*.* Additionally, designing learning in the context of culture is a specific strategy related to the curricular vision, knowledge of teaching, disposition, and professional practice of teachers that are adaptive to the environment familiar to students.

Educational progress comes from forward-looking studies that embrace students’ cultural advantages with diverse experiences of racism, poverty, trauma, and oppression. A dynamic learning environment reinforces culture, and the teacher respects the assets discovered in indigenous knowledge, values, and stories as a model of vitality and empowerment for all (Malia and Malone, 2017). Therefore, preservice teachers need to be equipped to use the indigenous environment to prepare a lesson plan. The impact is that they tend to survive as teachers in any environment, which affects sustainable education. This article discusses the various ways to equip preservice teachers to explore and use artifacts from indigenous communities to prepare their lesson plans. Subsequently, the solution is to observe the environment and appreciate the existing culture.

### 1.1 Preservice math teachers

Teachers are the spearhead of success in learning. Therefore, the Indonesian government trains them in ongoing learning efforts by helping them to build strong networks with their peers to improve their competence (Kyu et al., 2017; Sudja and Yuesti, 2017; Sumaryanta et al., 2019). Additionally, preservice teachers are properly equipped, and are agents of change who are trained to adopt the technology of indigenous knowledge during teaching and learning instructional planning (Kim and Baylor, 2008).

Preservice teachers need to be appropriately trained to become professionals with the ability to prepare learning instruction to help students master certain abilities and to harness their surrounding environment (Felder and Brent, 2005). It is essential to understand the environment to accommodate students’ prior education and their indigenous knowledge abilities. Preservice math teachers need to be observant in designing learning that accommodates indigenous knowledge.

### 1.2 The math environment of indigenous communities in education

Although mathematical truths hold everywhere and for everyone, this does not mean that math education should ignore learners’ individuality or the social and cultural context of education (Bishop, 1997). Hence, we need to do more than merely inform learners of these truths. We can find knowledge in the social context from the indigenous community, and also with math.

Mathematical concepts inherent and practiced in the indigenous community are known as ethnomathematics; this notion was first introduced by D'Ambrosio (Tutak et al., 2011). The term is derived from a combination of two words, “ethno” and “mathematics,” where the prefix indicates the sociocultural context, while the suffix is based on mathematical knowledge such as counting, comparing, sorting, classifying, designing, playing, weighing, and measurement (Katsap, 2018).

Ethnomathematics in learning encourages teachers and students to appreciate the diversity of knowledge (Powell, 2009). Further, Powell (2009) states that the context of community cultural material influences their knowledge and activities in the world. Students learn the importance of respecting their knowledge and others, and how all cultural backgrounds can interact with mathematical knowledge development. Powell's research (2009) shows examples of various forms in which mathematical knowledge can be coded differently from academic textbooks (school math textbooks).

Ethnomathematics, through indigenous knowledge, has a positive impact and is adopted in learning mathematics (i.e., the use of drama-mediated mathematical knowledge) (Stathopoulou et al., 2015), the integration of ethnomathematical folklore games in math learning (Fouze and Amit, 2018), the use of culturally based gender-relevant teaching (Mogari, 2017; Weldeana, 2015), and integrating ethnomathematics into an instructional approach in practice had the greatest mean gain in the acquisition of creative skills (Ogunkunle and George, 2015). Moreover, indigenous knowledge as material in math learning can make math interesting and valuable for underrepresented groups in increasingly diverse populations (Rosa and Orey, 2016). The use of indigenous knowledge tends to boost students' motivation in learning math, and reduces feelings of dissatisfaction and inequity (Verner et al., 2013). Moreover, it encourages respect for culture and math, and leads to improved learning achievement because it uses indigenous contexts. Culturally relevant pedagogy is one way to support learning among indigenous students (Abrams et al., 2013). Students’ indigenous knowledge naturally makes it easier to understand math, and tends to accept learning in a meaningful manner.

### 1.3 Preservice math teachers’ lesson plan for sustainable education

The design of a math lesson plan for sustainable education needs to be in accordance with the curriculum's learning outcomes and students' culture. Teaching students from remote areas requires collaborating with indigenous communities and designing lesson plans in accordance with their culture. They need to be involved in preparing learning instructions (Chinn, 2006, 2007, 2012; Lees, 2016; Owens, 2014). Accordingly, teachers need to interact with indigenous people, as they are better prepared to meet students' needs during learning activities (Lees, 2016).

Owens' (2014) research helped teachers, schools, and communities to implement appropriate and effective professional development, build partnerships between institutes and societies, revise teaching approaches and curricula, and to value cultural heritage and aboriginal knowledge. In other words, teachers’ interactions with indigenous people tend to alter their perceptions, skills, and abilities during teaching or learning activities.

In other studies, teachers and native Hawaiian instructors worked together on immersion science for five days. They met several times at rural schools, universities, and community sites, sharing programs and developing knowledge (Chinn, 2006). According to Chinn, long-term professional development that provides learning through cultural immersion, translation, and interdisciplinary instructions aids in establishing a practical community in which participants are taught as needed for the relevant growth of the community, place, and standards following the curriculum and pedagogy.

Chinn stated that preservice teachers carry out observations and interview indigenous teachers immediately when they arrive at these locations. This helps to map technologies to visualize and integrate indigenous-based Hawaiian knowledge with science inquiry; these are promising instructional strategies (Chinn, 2014).

The teacher designs the engagement of indigenous knowledge in learning math. Therefore, future math teachers need to be prepared to achieve the following: a) visualize math incorporated into the real world, b) investigate the mathematical ideas and practices of their pupils, and c) discover ways to incorporate it into the curriculum elements belonging to the sociocultural environment of the pupils in the classroom. However, it tends to positively motivate and increase the interest and curiosity of pupils toward math (Bonotto, 2001).

In some cases, preservice teachers that teach indigenous people tend to be either from developed or remote places. Assuming the teacher's hometown is an indigenous place, then learning instructions are designed by criticizing or evaluating his/her teaching and learning, integrating local knowledge into an established curriculum, or replacing it entirely and utilizing it to the advantage of the students (Vinlove, 2016). However, the teacher is not from a remote area, and learning instructions are designed by understanding the community through learning from the students and indigenous people, and asking the inhabitants for feedback and ideas concerning integrating local knowledge into the curriculum (Vinlove, 2016).

### 1.4 Ethics on seeking and drawing upon indigenous knowledge

Conducting research needs to follow ethics. Research involving humans and animals, both experimental and social research, should use ethics. Research ethics are concerned with minimizing harm, respecting autonomy, preserving privacy, and acting equitably (Hammersley, 2015). However, the commodification of indigenous knowledge is often acquired by less than ethical means. “The commodification of indigenous knowledge without consent, consideration, or compensation is another form of exploitation and marginalization of indigenous peoples” (Battiste, 2016).

However, well-intentioned, indigenous knowledge research is not enough to take indigenous knowledge without community or committee approval. The research should protect cultural groups’ assets, so research on indigenous knowledge requires university ethics committee approval by considering protection issues for the collective. Research that does not do so may contribute to the continued appropriation and plundering of indigenous culture, heritage, and knowledge (Battiste, 2016). Core ethical principles on research related to indigenous peoples and local communities are respect, the recognition of rights, responsibility as a scholar, mindfulness, participation, and mutual benefits (Tunón et al., 2016). Hence, research on indigenous knowledge must have the university committee approval and indigenous community agreements, respect, recognition of rights, responsibility as a scholar, mindfulness, participation, and mutual benefits.

## 2 Methodology

### 2.1 Research design

A qualitative approach was used due to its suitability for the study to reveal indigenous knowledge and assist the preservice teachers in designing math lesson plans. Preservice math teachers need to be trained to be able to explore indigenous knowledge, which is adopted as material to design learning instruction. Preservice teachers may take a course or engage in research activities as research assistants. Preservice teachers’ involvement in the discovery of indigenous knowledge can be achieved either individually or in small groups.

#### 2.1.1 Research participants

We chose two elderly individuals from the community as research participants. Additionally, according to the study's purpose, we involved preservice math teachers—who are members of the indigenous community, from the border of Yogyakarta and Central Java—as research assistants. In addition, the indigenous community approved of this study's activities. We gained university committee approval from the Institute of Research and Community Service Universitas PGRI Yogyakarta and indigenous community agreements from Beteng hamlet, Jatimulyo, Giri Mulyo, Kulon Progo.

#### 2.1.2 The data collected

The exploration of indigenous knowledge was achieved by observing and interviewing the indigenous community (Alangui, 2010; Chinn, 2014; Vinlove, 2016). Therefore, artifacts were observed, and elderly indigenous people were interviewed to acquire indigenous knowledge, which was utilized as material to design learning instructions. Prior to that, the researchers clarified that the data collected would be used to design math lesson plans.

Data were gathered from the indigenous community situated at the border of Yogyakarta and Central Java, Indonesia. The area is mountainous, and the inhabitants have some particular indigenous artifacts, namely*, kentongan* (a tool used to summon residents, send out danger signals, make announcements, etc.), *padasan* (a tank for water made of clay, which is used for ablutions), *tapih* batik (which serves as fabric for skirts), and so on.

Observation was carried out to identify the artifacts used. We observed and took pictures to document the artifacts. In addition, daily activities were also witnessed, while interviews were carried out to confirm the activities and objects utilized by the inhabitants. The length of each interview was approximately 30 min per day for two days. We performed a member-check for data triangulation. The interviews were recorded and transcribed. The questions included:

- The history and usability of artifacts
- The artifact creation process
- The size of each artifact

During the data collection, we involved preservice math teachers as research assistants. Their role was to document and record the interview activities. They also took pictures, observed, and measured the artifacts under study.

The indigenous knowledge acquired was employed to develop a math lesson plan; it also served as material in the learning design used. Indigenous knowledge is either in the form of daily activities, habits, or artifacts.

### 2.2 The data analysis

A qualitative procedure was employed for data analysis. This analysis generated interpretations of data or mathematical values that exist in indigenous knowledge. The analysis was also linked to the relational ideas of the artifacts and the math curriculum content. The results of the analysis were used to develop a math lesson plan.

The preservice teachers engaged in the data analysis. They were involved in discussions on math object-related indigenous artifacts. They also engaged in developing lesson plans. In other words, the data analysis involved the indigenous community.

## 3 Results

### 3.1 Indigenous artifacts and mathematical objects related to them

A form of ethnomathematics study is conducted in terms of artifacts that tend to be discovered in various aspects of people's lives. The basic needs of humans are food, clothing, and shelter. Based on the aspect of food, they need to cook and eat. Therefore, artifacts are required in carrying out processes, going places, etc. In accordance with the context of clothing, there are certain clothes that indigenous, such as *tapih*, which serves as fabric for skirts worn by elderly women. Additionally, there are various kinds of buildings. One of the buildings is the indigenous home. Indigenous (Javanese) houses have various styles depending on the strata (i.e., a balanced roof with right and left sides for a common house, *limasan* for a middle house, and *joglo* for a luxury house).

The homes of indigenous people are built according to numerous rules (i.e., regarding manufacturing time, measurement, and other elements). Based on the information obtained from one of the villagers in the mountainous region:

*Nek damel griyo niku wonten petunganipun, kapan le pasang, jumlah usuke piro. Kabeh eneng petungane.* [There are many considerations in building a house, i.e., when is the right time to build, the number of ribs. All of that has a calculation.]

(Interview excerpt, March 11, 2020)

**Figure 1:**
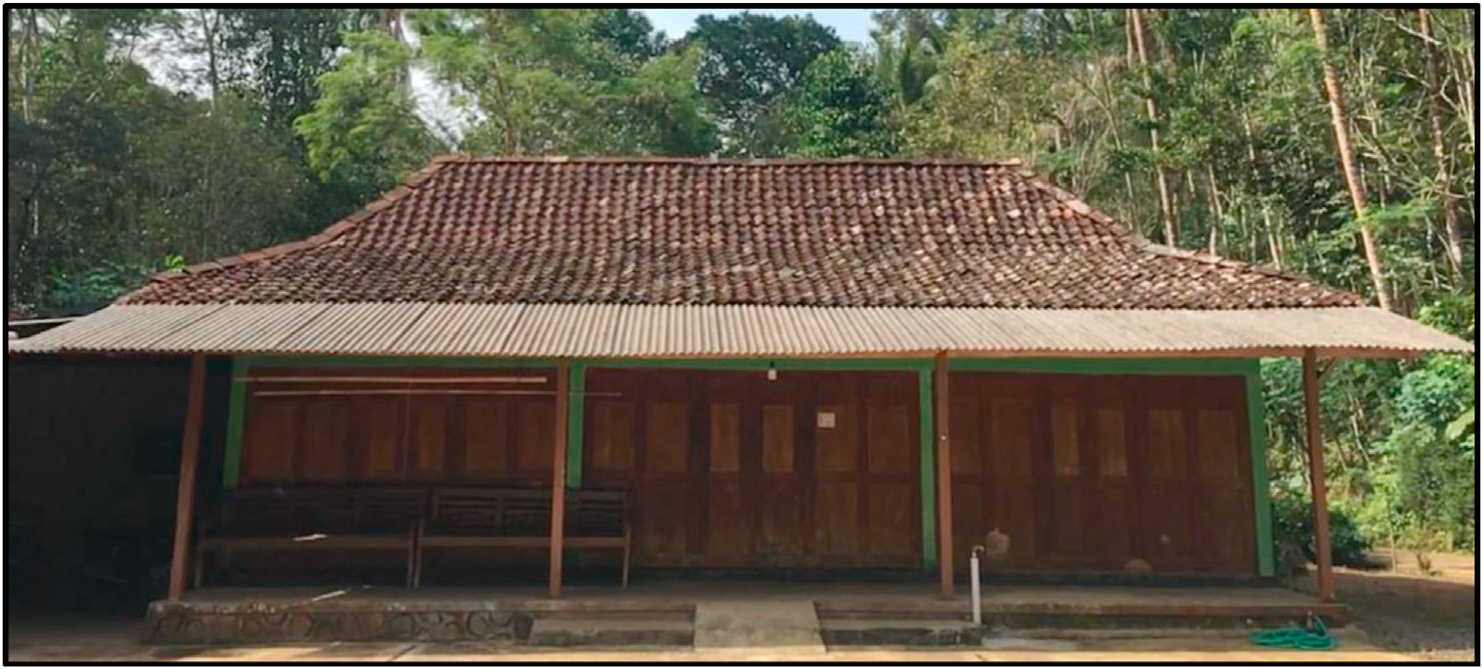
An indigenous home on the border between Yogyakarta and Central Java, Indonesia.

Based on traditional elderly individuals, many mathematical values can be derived. However, the mathematical values that we will discuss here are related to geometric shapes. A picture of an indigenous house at the border of Yogyakarta and Central Java, Indonesia is shown in Figure 1.

**Figure 2:**
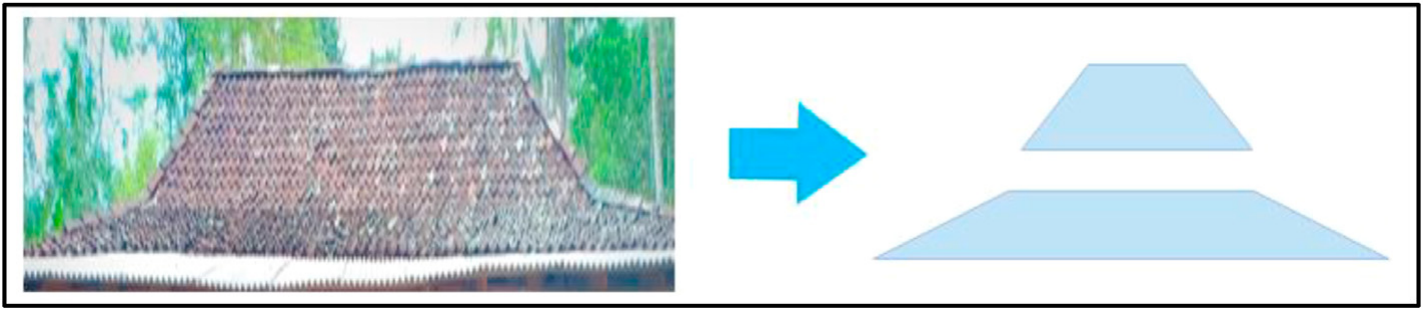
The roof of the indigenous house and related mathematical objects.

The roofs of the houses in the area are either made of tiles or straw. The tiles are arranged in a certain pattern to form a roof frame fabricated into an isosceles trapezoid. This, of course, is used as an example in teaching the concept of the trapezoid, as well as several other objects. The roof of the house and the related mathematical objects are shown in Figure 2. In addition, the roof of the house is used as an enrichment material in the application of problems encountered on a daily basis. However, the problem, in this case, is discovering the amount of wood needed to make the roof frame.

In addition to building homes, other artifacts are solids used for purification before praying. One of them is the *padasan*, an object made out of clay that serves as a water reservoir for the Javanese. Based on the information obtained from one villager in the mountainous region:

*Padasan niki biasane diengge bebersih, bisa cuci tangan, wudlu, mandi juga bisa.* [Padasan is usually used for sacred practices, such as the washing of hands, ablutions, and for bathing.]

(Interview excerpt, March 11, 2020)

This implies that it serves as a tap and is usually placed in front of the house or beside the well.

**Figure 3:**
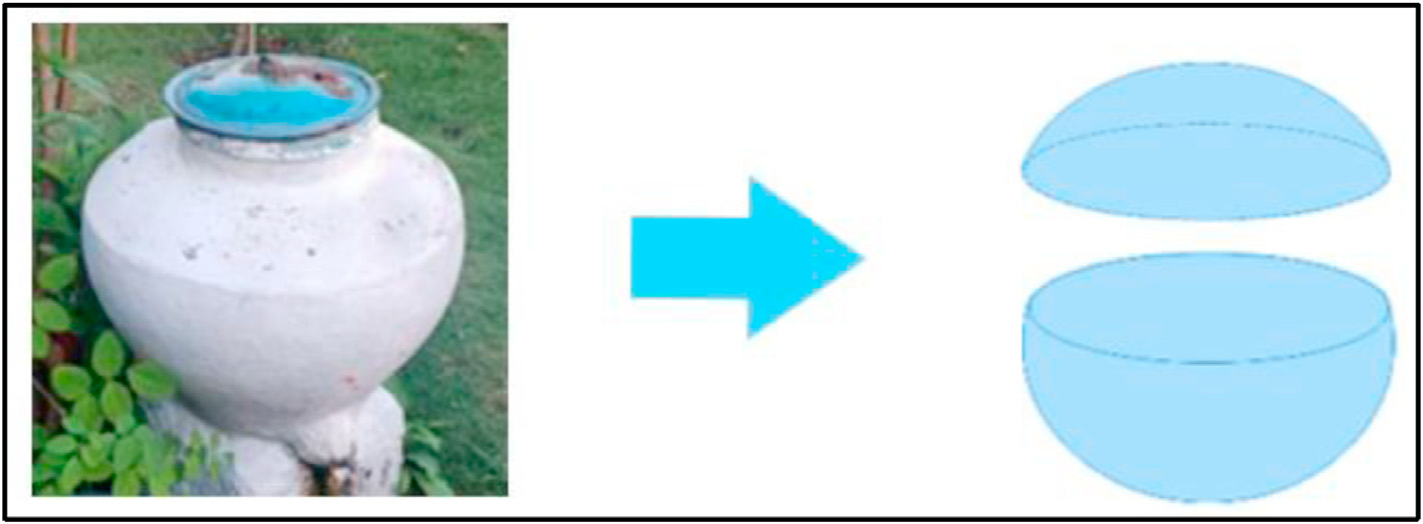
The *padasan* and related mathematical object.

This led to the observation of solids that contain the combined mathematical values of two truncated spheres. Learning that utilizes material that deals with the application of daily problems—for example, how many liters or buckets of water does a *padasan* contain, etc.—enriches learning. The *padasan* is regarded as a metaphor or close analogy with the truncated sphere. Materials concerning the concept of the truncated sphere are also carried out on this basis. The *padasan* artifact and related mathematical object are displayed in Figure 3.

In addition to the necessities of life, humans, as social beings, also need to interact with one another. The civic needs of individuals are dependent on the current rules of society and various artifacts. One of them is called the *kentongan*, which is usually located on the *cakruk* (a kind of security post); it is a community communication tool made of wood. In accordance with the information from one of the villagers in the mountainous region:

*Kentongan niki biasane diengge titir nek ono bahaya, ono gempa, ono maling* [Kentongan is usually used for warning against danger, tremors, and theft].

(Interview excerpt, March 11, 2020)

**Figure 4:**
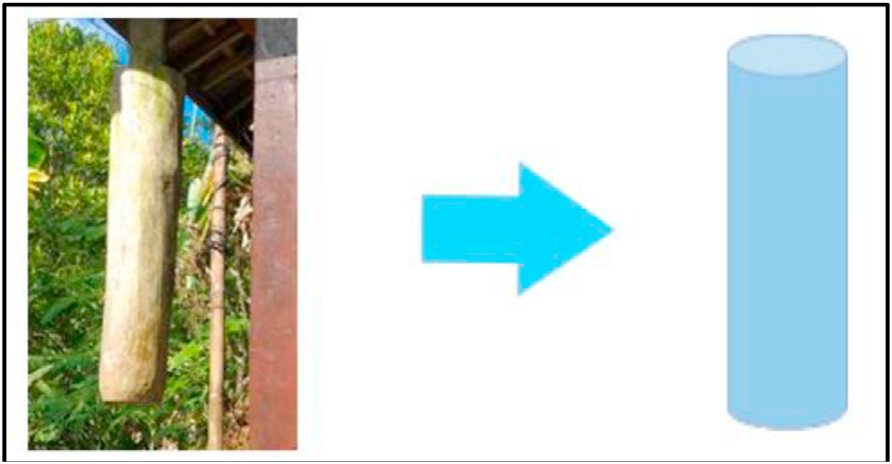
The *kentongan* and related mathematical object.

This means that the *kentongan* is a communication tool used to inform people of any sign of possible danger, to gather inhabitants whenever there is an urgent meeting or community service, etc. The *kentongan* artifact and the related mathematical object are depicted in Figure 4. It has a tube-like shape that is used in learning math, both in the material presented at the beginning of this context and in relation to daily activities.

### 3.2 Exploring learning materials from indigenous artifacts

We need to be attentive to the mathematical knowledge of indigenous people. Based on the numerous examples of the artifacts utilized in math learning, we chose 3 examples, namely, the roof of the house, the *padasan*, and the *kentongan*. Artifacts, mathematical objects, and learning materials derived from the ethnomathematics of the inhabitants on the border between Yogyakarta and Central Java are summarized in Table 1.

**Table 1:** Indigenous artifacts as learning materials.

Indigenous artifact	Mathematical object	Learning material
Roof	Trapezoid	**Concept** Concept of the trapezoid
		**Math problem** The area of the trapezoid
*Padasan*	Truncated sphere	**Concept** Concept of the sphere
		**Math problem** The volume and surface area of the truncated sphere
*Kentongan*	Tube	**Concept** Concept of the tube
		**Math problem** The surface area of the tube

#### 3.2.1 Math lesson plan with “roof” context

Construction of the lesson plan requires an analysis of the curriculum. In accordance with the 7^th^ grade mathematics learning curriculum in Indonesia, the artifact “roof” can be adopted at the beginning of learning as a context related to daily life (see Tables 2 and 3).

Relates to the circumference formula and area for various types of quadrilaterals (square, rectangle, rhombus, parallelogram, trapezoid, and kite) and triangle.

(Permendikbud, 2018)

**Table 2:** Design of learning activity 1.

Indigenous artifacts	Description of activity	Conjecture of student learning
Roof	Teacher poses an example of the trapezoid in daily life (roof) through the display on the screen. The teacher refers to Javanese houses that are common, *limasan* for the middle, and *joglo* for the luxurious house.	Some students will imagine the form of a trapezoid from the situation.
	Teachers ask students to look for another trapezoid example.	Some students will look for another trapezoid on daily life.

**Table 3:** Design of learning activity 2.

Indigenous artifacts	Description of activity	Conjecture of student learning
Roof	The teacher poses a contextual problem in the roof area. Roof area to estimate the minimum amount of tile needed. Calculating the number of tiles based on the roof area truly helps us to prepare the budget design. The teacher gives clues and asks key questions to direct students' minds in inquiry and to find the solutions: a. How many tiles are on the roof? Count them! b. Draw the roof on the paper, cut, and flip the triangle. Conclude your findings!	Some students will think, based on the situation, about how to count the tiles. Some students will discuss the solution to the problem, such as drawing on paper, counting, and discovering the formula.
	The teacher asks two groups of students to present the results of their work.	Some students will pay attention to the exposure.
	The teacher considers unanswered questions and helps students to make connections back to quadrilaterals. The teacher evaluates the process.	

Based on the curriculum, the lesson plan of math learning for 7^th^-grade students regarding the trapezoid through the roof is presented as follows.

In addition, the roof-related trapezoidal contextual problem is presented. According to the 7^th^-grade math learning curriculum in Indonesia:

Solve contextual problems related to the area and circumference of quadrilaterals (square, rectangle, rhombus, parallelogram, trapezoid, and kite) and triangle.

(Permendikbud, 2018)

Based on the curriculum, the lesson plan of math learning for the 7^th^ grade regarding the trapezoid through the roof is presented as follows.

#### 3.2.2 Math lesson plan in the “padasan” context

Further, the construction of a lesson plan through the *padasan* can be done as a context of the truncated sphere. In accordance with the 9^th^-grade math learning curriculum in Indonesia, the artifact “*padasan*” can be adopted at the beginning of learning as a context related to daily life (see Tables 4 and 5).

Generalize the surface area and volume of various curved side spaces (tube, cone, and sphere).

(Permendikbud, 2018)

**Table 4:** Design of learning activity 3.

Indigenous artifacts	Description of activity	Conjecture of student learning
*Padasan*	The teacher poses an example of a sphere and truncated sphere in daily life through the display on the screen. The teacher refers to the *padasan* that indigenous people usually use for sacred practices, such as washing their hands and ablutions.	Some students will imagine the form of a sphere and truncated sphere based on the situation.
	Teachers ask students to look for another sphere and truncated sphere example.	Some students will look for the other sphere and truncated sphere on the daily life.

**Table 5:** Design of learning activity 4.

Indigenous artifacts	Description of activity	Conjecture of student learning
*Padasan*	The teacher poses a contextual problem of the truncated sphere area. The truncated sphere area of the *padasan* is needed to estimate the minimum number of paint cans needed.	Some students will think based on the situation, how to estimate the minimum number of paint cans needed.
	The teacher give clues and asks key questions to direct the students' minds in inquiry and to find the solutions: Compare the area of the truncated sphere with the sphere. How is the comparison? Hence, could you make a conclusion from the surface area of the truncated sphere and the sphere?	Some students will discuss the solution to the problem, such as making comparisons with geometric shapes and discovering the formula.
	The teacher asks two groups of students to present the results of their work.	Some students will pay attention to the exposure.
	The teacher considers unanswered questions and helps students to make connections back to the surface area of the sphere. The teacher evaluates the process.	

Based on the curriculum, the lesson plan of math learning for 9^th-^grade students regarding the truncated sphere through the *padasan* is presented as follows.

In addition, the roof-related sphere contextual problem is presented. According to the 9^th^-grade math learning curriculum in Indonesia:

Solve contextual problems related to surface area and volume of arcing (cylindrical, conical, and spherical side space), as well as the combination of several curved sided spaces.

(Permendikbud, 2018).

Based on the curriculum, the lesson plan of math learning for the 9^th^ grade regarding the truncated sphere through the *padasan is* presented as follows.

#### 3.2.3 Math lesson plan in the “kentongan” context

Moreover, the construction of the lesson plan through the *kentongan* can be done as a context of the tube. In accordance with the 6^th^-grade math learning curriculum in Indonesia, the artifact “*kentongan*” can be adopted at the beginning of learning as a context related to daily life (see Tables 6 and 7).

**Table 6:** Design of learning activity 5.

Indigenous artifacts	Description of activity	Conjecture of student learning
*Kentongan*	The teacher poses an example of the tube in daily life (*kentongan*) through the display on the screen. The teacher refers to the *kentongan,* which is used to inform the people of any sign of possible danger, to gather the inhabitants whenever there is an urgent meeting or community service, etc. (communication tool)	Some students will imagine the form of the tube based on the situation.
	The teachers ask students to look for another tube example.	Some students will look for another tube on daily life.

**Table 7:** Design of learning activity 6.

Indigenous artifacts	Description of activity	Conjecture of student learning
*Kentongan*	The teacher poses a contextual problem of the tube area as an example: The tube area of the *kentongan* is needed to estimate the minimum number of paint cans needed.	Some students will think based on the situation, how to estimate the minimum number of paint cans needed. Some students will discuss the solution to the problem, such as making comparisons with geometric shapes and discovering the formula.
	The teacher asks two groups of students to present the results of their work.	Some students will pay attention to the exposure.
	The teacher considers unanswered questions and helps students to make connections back to the surface area of the tube. The teacher evaluates the process.	

Compare prism, tube, pyramid, cone, and sphere (Permendikbud, 2018).

Based on the curriculum, the lesson plan of math learning for the 6^th^-grade students regarding the tube through the *kentongan is* presented as follows.

In addition, the *padasan* is related a tube contextual problem. According to the 6^th^ grade math learning curriculum in Indonesia:

Identify prism, tube, pyramid, cone, and sphere.

(Permendikbud, 2018).

Based on the curriculum, the lesson plan of math learning for the 6^th^ grade regarding the tube through the *kentongan is* presented as follows.

All of the presented examples are alternatives that can adopt artifacts for use in the math lesson plan. The lesson plan, which elaborates on indigenous people's artifacts, has the potential to connect culture and mathematics.

## 4 Discussion

Sustainable education in remote areas depends on the creativity of the teachers in preparing lesson plans, such as employing artifacts from indigenous communities. The artifacts of indigenous people need to be incorporated into math learning materials. The use of familiar objects (such as indigenous artifacts) makes students feel that their culture is valued. In addition, it is a more meaningful way of learning math in accordance with the cultural context. Elaborating on the artifacts of indigenous people has the potential to connect culture and math.

Indigenous artifacts have a lot of potential that can be used in math learning. Teachers or preservice teachers should master it. Therefore, it is urgent to involve preservice teachers in revealing their culture because they can incorporate their culture into their lesson plan, facilitating their students' meaningful math learning. Hence, preservice teachers need to be trained to prepare lesson plans with environmental materials that are familiar to students. Preservice teachers need to be trained to be adaptive, creative, and innovative to promote sustainable education in remote areas. The research involves preservice teachers as assistants in interviews, observations, and measurements. Additionally, preservice teachers were invited to discuss the interpretation of the artifacts’ mathematical values and how these artifacts can be used in designing lesson plans. The concept of involving indigenous knowledge in math learning for both trained and preservice teachers needs to be included in ethnomathematically-based education courses (Dawson, 2013; Iluno and Taylor, 2013; Verner et al., 2013). Training also needs to be conducted through an ethnomathematics study, that involves indigenous people, because such an examination would reveal the cultural, social, and political dimensions of math education (Knijnik, 2002).

The challenge aimed at exploring the indigenous knowledge of learning materials is to portray mathematical ideas from non-Western perspectives (Alangui, 2010). However, there is a need to be thorough and attentive to people's mathematical knowledge. Although the interpretation of mathematical values in indigenous knowledge requires particular abilities, it is beneficial to learning. It is more valuable and meaningful to follow the context with which the students are already familiar.

Examples are artifacts that are made and used in remote areas on the border between Yogyakarta and Central Java, Indonesia such as the trapezoid of the roof, the truncated sphere, and the tube-like shape of the *padasan* and *kentongan* materials, respectively. There are still many other artifacts that may be utilized in learning math in such areas. Further studies are needed to investigate each of these artifacts’ mathematical values in greater depth, as discussed in ethnomathematics research.

The explanation of learning materials from the artifacts discovered along the border between Yogyakarta and Central Java shows that artifacts can be adopted as materials in math lesson plans. Elaborating on indigenous people's artifacts potentially links cultures and math (Civil, 2014; Naresh and Kasmer, 2018). It also leads to more meaningful math learning (Sharma and Orey, 2017). Preservice teachers need to be accustomed to preparing materials for lesson plans in accordance with the environment that is familiar to the students. They also need to be adaptive, creative, and innovative in remote areas to produce sustainable education.

### 4.1 Limitations of this study

Several limitations must be considered when interpreting the reported results and conclusions. First, there were only two preservice teachers involved in this study. Therefore, the observation is limited to certain artifacts. The other artifacts cannot be discussed yet. Second, not all artifacts are suitable or the same as objects in math. The artifacts are similar to mathematical objects. More so, the intended use of culture highlights cultural values. Nevertheless, the emphasis is placed on contextual problems from indigenous communities.

### 4.2 Recommendations for future research

Considering that ethnomathematics studies regarding Javanese indigenous knowledge have increased in the last few years, this study's repetition is recommended in the future, as many more studies will be published in the coming years. Research on ethnomathematics studies in less examined educational stages, such as early childhood, higher education, or preservice teachers and teacher training, may also be a substantial field of study.

## 5 Conclusion

Indigenous artifacts might be similar to mathematical objects. It is regarded as a metaphor or close analogy with a mathematical object. Therefore, indigenous artifacts have an opportunity for sustainable education in remote areas. Hence, teachers or preservice teachers need to integrate local knowledge into their instruction.

Involving preservice teachers in ethnomathematics studies improves their capabilities for preparing lesson plans relevant to the student environment for sustainable education. This methodology develops preservice teachers' education or teacher training and empowers indigenous knowledge. Further, preservice teachers, who are also members of indigenous communities, would engage in understanding the mathematical values that exist in their indigenous culture. The implication of this is the increasing number of professional teacher candidates who are agents of change to be more adaptive wherever they are to teach. Thus, the quality and quantity of professional teachers’ distribution is more spread evenly.

## Declarations

### Author contribution statement

Niken Wahy Utami: Conceived and designed the experiments; Performed the experiments; Analyzed and interpreted the data; Contributed reagents, materials, analysis tools or data; Wrote the paper.

Suminto A. Sayuti, Jailani: Conceived and designed the experiments; Analyzed and interpreted the data; contributed reagents, materials, analysis tools or data; Wrote the paper.

### Funding statement

This work was supported by the Team of Acceleration of a Reputable International Journal and the Institute of Research and Community Services Universitas PGRI Yogyakarta.

### Data availability statement

Data included in article/supplementary material/referenced in article.

### Declaration of interests statement

The authors declare no conflict of interest.

### Additional information

No additional information is available for this paper.
